# Galectin-3 as the Prognostic Factor of Adverse Cardiovascular Events in Long-Term Follow up in Patients after Myocardial Infarction—A Pilot Study

**DOI:** 10.3390/jcm9061640

**Published:** 2020-05-29

**Authors:** Przemysław Święcki, Robert Sawicki, Małgorzata Knapp, Karol Adam Kamiński, Katarzyna Ptaszyńska-Kopczyńska, Bożena Sobkowicz, Anna Lisowska

**Affiliations:** Department of Cardiology, Medical University of Białystok, 15-276 Białystok, Poland; przemo933@wp.pl (P.Ś.); r-sawicki@o2.pl (R.S.); malgo33@interia.pl (M.K.); fizklin@wp.pl (K.A.K.); kasia.ptaszynska@op.pl (K.P.-K.); sobkowic@wp.pl (B.S.)

**Keywords:** galectin-3, coronary artery disease, atherosclerosis, carotid arteries

## Abstract

Galectin-3 (Gal-3) is a new independent risk factor in the development and severity of coronary artery disease (CAD). The aim of the study was to evaluate whether Gal-3 concentration has prognostic value and if it reflects the progression of atherosclerosis in carotid arteries in patients with CAD after acute myocardial infarction (AMI). The analysis included 110 patients who were hospitalized due to AMI, treated with primary coronary intervention (PCI) and further attended a follow-up visit, and 100 healthy volunteers. The Gal-3 concentration and carotid ultrasound were evaluated at baseline and on a follow-up visit. We found that the Gal-3 concentration in the group with hyperlipidemia decreased during the observation (10.7 vs. 7.9 ng/mL, *p* = 0.00003). Patients rehospitalized during follow up had higher concentration of Gal-3 in the acute phase of myocardial infarction (MI) (10.7 vs. 7.2 ng/mL, *p* = 0.02; 10.1 vs. 8.0 ng/mL, *p* = 0.002, respectively). In the group of patients who had none of the following endpoints: subsequent MI, PCI, coronary artery bypass grafting (CABG) or stroke, there was a decrease in Gal-3 concentration at the follow-up visit. Parameters affecting the frequency of a composite endpoint occurrence are: the presence of atheromatous plaque in the carotid artery (*p* = 0.017), Gal-3 (*p* = 0.004) and haemoglobin (*p* = 0.03) concentration. In multivariate analysis, only Gal-3 concentration higher than 9.2 ng/mL at discharge was associated with a nine-fold increase of risk of composite endpoint occurrence (*p* = 0.0005, OR = 9.47, 95% CI 2.60–34.45). A significant decrease in Gal-3 concentration was observed in the group of patients after AMI without the endpoint occurrence during observation.

## 1. Introduction

Galectin-3 (Gal-3) is a macrophage- and endothelium-derived mediator actively involved in the regulation of many aspects of inflammatory cell behaviour. Gal-3 is a molecule well-known in cancer [1,2], in the vascular system associated with inflammation, venous diseases [3] and cardiac fibrosis in patients with heart failure (HF). A higher concentration of Gal-3 is associated with an increased risk for a HF incident and mortality [4,5,6]. Some results suggest that Gal-3 production is involved in the developmental process of atherogenesis [7]. They also suggest a unique protective role of Gal-3 in the uptake and effective removal of modified lipoproteins with concurrent downregulation of proinflammatory pathways responsible for atherosclerosis initiation and progression [8]. Higher expression of Gal-3 in macrocalcified plaques was observed [9]. It is probable that the inhibition of Gal-3 causes a decrease of atherosclerosis [10]. According to research by Tsai et al. [11], patients with unstable coronary artery disease (CAD) had higher plasma Gal-3 concentration compared to stable subjects. Moreover, the circulating level of Gal-3 was significantly higher in patients with a myocardial infarction (MI) than in healthy control subjects [12]. Whereas others [13] did not observe a significant difference in Gal-3 concentration between these groups of patients. Recently published data suggest that Gal-3 is an independent risk factor of a coronary artery disease occurrence [12]. Furthermore, the elevated concentration of circulating Gal-3 was the strongest independent predictor of the combined 30-day major adverse clinical outcome (MACO) (defined as advanced HF or 30-day mortality) [11]. Similarly, in a mid-term observation, the Gal-3 concentration, marked during 24 h following an acute MI, was observed to be an independent predictive indicator of the increased risk of all-cause mortality in patients after MI [12]. However, other researchers observed that Gal-3 is not associated with an outcome in older patients with an advanced chronic systolic HF of ischemic etiology when adjusting for N-terminal pro-brain natriuretic peptide (NT-pro-BNP) and may, therefore, have limited use in the prognostication [14]. Additionally, in another study, a baseline Gal-3 concentration did not predict the outcome after three months following primary coronary intervention (PCI) in an acute MI. Therefore, the role of Gal-3 as a prognostic marker during long-term follow up in patients after MI is still debatable. The aim of the study was to analyse whether the Gal-3 concentration assessed during at least a 24-month period following MI has any prognostic value, and whether it reflects the progression of atherosclerosis and correlates with the intima-media thickness (IMT) complex and the presence of atheromatous plaque in carotid arteries in this group of patients during long-term observation.

## 2. Material and Methods

### 2.1. Selection of the Study Population

The study group included patients with acute coronary syndromes (ACS) who were hospitalized at the Department of Cardiology between 2012 and 2014 and underwent invasive procedures—a coronary angiography and angioplasty in the acute phase of MI—and further, they attended a follow-up visit. The inclusion criteria were: age between 18 and 75 years, coronary angiography as the procedure in ACS, a transthoracic echocardiography (TTE) and an examination of carotid arteries with the use of Doppler ultrasound. Patients who had ACS complicated by cardiogenic shock and acute heart failure were excluded from the study. Selected biochemical and clinical risk factors of the progression of atherosclerosis were evaluated. Finally, 110 patients were enrolled: 66 patients diagnosed with ST-elevation myocardial infarction (STEMI) and 44 patients with non-ST-elevation myocardial infarction (NSTEMI). The characteristics of the study group are presented in Table 1.

An informed consent by all participants examined for the report was obtained. The study project was approved by the Local Bioethics Committee (R-I-002/411/2018).

### 2.2. Control Group

The control group included 100 healthy volunteers selected according to sex and age (average age 61.5 ± 7.9 years) who were subjected to their standard periodical checkups at the University Hospital Departments. Detailed control group characteristics have been published previously [12]. The standard analysis has shown that the control group had significantly lower systolic blood pressure (BP), lower concentrations of total cholesterol, low density lipoprotein (LDL) and glucose, a higher concentration of high density lipoprotein (HDL), a significantly higher value of glomerular filtration rate (GFR) as well as a significantly higher left ventricular ejection fraction (EF); they smoked cigarettes less often compared to the MI group (Table 1).

### 2.3. Biochemical Evaluation

The venous blood samples were obtained within the first 24 h after patient admission to the hospital. The blood was drawn to clot in a closed system of Monovette type (SARSTEDT, Nümbrecht, Germany) for further analyses of troponin I, C-reactive protein (CRP), total cholesterol, LDL cholesterol, HDL cholesterol, triglycerides, glucose, creatinine and Gal-3 concentration. Biochemical parameters were analysed within two hours following blood sample collection. A GFR was calculated using the Cockroft–Gault formula. The blood drawn for determination of Gal-3 was incubated for 30 min and then centrifuged for 15 min with the speed 1000× *g*. The obtained serum was stored at −70 °C until the designation was performed. The serum samples were collected over a one-year period from the beginning of January 2016 to January 2017 (minimum time 6 months, maximum time 18 months and mean time 12 months). The collection tube for the serum did not require anticoagulant. The measurements were performed strictly according to the manufacturer’s instructions in a certified laboratory. Serum Gal-3 levels were measured in duplicate using a commercially available enzyme-linked immunosorbent assay (ELISA) Human Gal-3 Quantikine Kit (catalog no.: DGAL30, R&D Systems, Minneapolis, MN, USA). The analysis was done with the Epoch BioTek microplate reader (Winooski, VT, USA) and allowed for the detection and estimation of the tested protein concentration with the use of appropriate antibodies: monoclonal or polyclonal. The measurements were done strictly according to the manufacturer’s instructions. The method was based on the antigen–antibody sets whereas their detection was possible due to an enzymatic reaction.

### 2.4. Echocardiography

Standard TTE with heart chambers measurement, left ventricle walls thickness, walls contractility and an EF were performed with an ultrasound device (Philips iE33, Bothell, WA, USA) with the use of a transthoracic probe working with harmonic imaging within the frequency range of 1.6–3.2 MHz. Further, the left ventricular EF was estimated visually and by Simpson’s method. Left ventricular EF ≥50% has been accepted as a normal value.

### 2.5. Coronary Angiography

Angiographic radial access (98%) with the use of a 6F catheter and standard projections were performed in the study population. A significant coronary lesion was defined as a lumen narrowing of more than 50% and further the study group was divided into one-, two- and three-vessel disease subgroups.

### 2.6. Doppler Ultrasonography of the Carotid Arteries

An examination of the common carotid artery (CCA) and the carotid bulb (CB) was performed with an ultrasound device (Philips iE33) equipped with a 3–11 MHz linear-array high-resolution transducer, using dedicated software for a B-mode analysis, as described previously [12]. Further analysis included the following parameters: IMT and the presence of atheromatous plaques in the vessel. The CCA was scanned along a 10 mm-long segment from the carotid bulb. In some patients, images of the internal carotid artery (ICA) were of inadequate quality, resulting in the ICA being excluded from further analysis. IMT measurements were made for the distal wall because the IMT assessment for the proximal wall was complicated by the higher echo density of the adventitia than of the media and intima. The distance between the first clearly delineated bright line (the lumen/intima interface) and second bright line (the media/adventitia interface) of the distal wall was measured as the IMT. For each analysed segment of the vessel, two IMT measurements were taken and an average calculated. The maximum IMT measurements were further used in the calculations. A plaque diameter was defined as an IMT of more than 1.5 mm [15,16].

### 2.7. Follow-Up Visit

The average follow-up observation was done after 41.3 ± 9.6 months. During this visit, patients completed a questionnaire including risk factors, pharmacotherapy, cardiovascular events they underwent (including subsequent MI, stroke, carotid and/or lower limb symptomatic atherosclerosis), another hospitalization or re-revascularization. They had the standard echocardiography, IMT complex ultrasound with the evaluation of atherosclerotic plaque in carotid arteries. Moreover, patients’ blood samples were obtained to assess the Gal-3 concentration.

### 2.8. Statistical Analysis

The mean values and standard deviations for quantitative variables were calculated, as well as the quantitative and percentage distribution for qualitative variables. To compare the groups, the statistical analysis for variables of a normal distribution, estimated using the Kolmogorov compatibility test, was done with the use of the unpaired Student’s *t*-test and the Mann–Whitney test for variables inconsistent with a normal distribution. A Pearson’s correlation coefficient was calculated for categorical variables of a normal distribution, and a Spearman’s correlation coefficient for variables with the distribution other than normal. The comparison of qualitative variables was done using the Chi-square test. A *p*-value of <0.05 was considered statistically significant. A multivariate analysis of the examined parameters has also been performed with the use of the model of multilogistic regression analysis using a stepwise approach. The changes of Gal-3 concentration were analysed in individual subgroups of patients at the two time points; analysis of variance (ANOVA) with repeated measurements was used. A *p*-value of <0.05 was considered statistically significant.

The statistical analysis was performed with Statistica 10.0 PL software (StatSoft Inc, Tulsa, OK, USA).

## 3. Results

### 3.1. Characteristics of the Study Group at Baseline and on the Follow-Up Visit

Clinical characteristics of the MI study group at the beginning of the study are presented in Table 1. Patients with NSTEMI differed considerably from STEMI patients with regard to sex; women were more often diagnosed with NSTEMI, while men were diagnosed more often with STEMI (*p* < 0.005). On admission, NSTEMI patients had a significantly lower concentration of glucose (*p* < 0.05) and creatinine. Clinical characteristics of the MI study group after follow up are presented in Table 2. During the follow-up period (that was on average 41.3 months), no significant differences in Canadian Cardiovascular Society (CCS) scale and New York Heart Association (NYHA) scale class between STEMI and NSTEMI patients were observed. However, STEMI patients had a statistically lower EF (45.6 ± 9.9% vs. 49.5 ± 9.8%, *p* = 0.04). In the NSTEMI group, four patients (9.1%) had a next MI, whereas there were no further MIs in the STEMI group (*p* = 0.005). Active smokers were more prevalent in the STEMI group (*p* = 0.04). Furthermore, they were more often treated with angiotensin-converting enzyme inhibitors (ACEIs) (*p* = 0.04).

Further analyses did not reveal differences between STEMI and NSTEMI patients in frequencies of rehospitalization (for both cardiac- and non-cardiac causes), subsequent revascularization and the occurrence of other adverse vascular events (stroke, symptomatic carotid and/or lower limb atherosclerosis) (Table 2).

### 3.2. Gal-3 Concentration

#### 3.2.1. At Baseline

Concentrations of Gal-3 determined in the acute phase of MI were significantly higher in the study group (STEMI and NSTEMI patients) as compared to the controls—median 7.8 ng/mL (5.6–10.2) and 7.6 ng/mL (6.4–9.8) vs. 5.6 ng/mL (4.1–7.2) (*p* = 0.00001), respectively. There were no differences between Gal-3 concentrations in the STEMI and NSTEMI groups. We showed positive correlation of the Gal-3 concentration with creatinine (r = 0.9, *p* = 0.02) and negative correlation with GFR (r = −0.3, *p* = 0.02).

#### 3.2.2. On the Follow-up Visit

The Gal-3 concentration was 8.2 ng/mL (7.0–9.7) in STEMI and 8.5 ng/mL (6.9–10.7) in the NSTEMI group, and it did not differ significantly from concentrations observed in the acute phase of MI (*p* = 0.53). We observed positive correlation of the Gal-3 concentration with IMT complex values in CCA (*r =* 0.3, *p* = 0.001) and the presence of the atheromatous plaque in CCA and CB (*r =* 0.28, *p* = 0.002; *r =* 0.8, *p* = 0.04, respectively). The Gal-3 concentration correlated negatively with EF (*r =* −0.9, *p* = 0.03).

### 3.3. Gal-3 Concentration and Cardiovascular Risk Factors

The baseline analysis showed higher concentration of Gal-3 in patients diagnosed with hyperlipidemia (44 patients, 40%) compared to patients without lipid disorders (66 patients, 60%) (14.7 ± 6.8 ng/mL vs. 10.7 ± 4.2 ng/mL, *p* = 0.01). However, the concentration of Gal-3 in patients with hyperlipidemia decreased significantly during the observation period (14.7 ± 6.8 ng/mL vs. 8.7 ± 2.4 ng/mL, *p* = 0.00003). The presence of diabetes mellitus and hypertension remained without significant influence on the concentration of Gal-3 at baseline and on the follow-up visit.

### 3.4. Gal-3 Concentration and Advancement of CAD

At baseline, there were no differences between the concentration of Gal-3 among patients with a one-, two- and three-vessel coronary artery disease. During the observation, a significant decrease in Gal-3 concentration was observed only in patients with single-vessel disease (12.3 ± 7.0 ng/mL vs. 8.2 ± 2.1 ng/mL, *p* = 0.007).

### 3.5. Gal-3 Concentration and Carotid Atherosclerosis

At baseline, a significantly higher Gal-3 concentration was observed in the group of patients with atherosclerotic plaques in carotid arteries vs. patients without carotid atherosclerosis (14.7 ± 7.2 ng/mL vs. 10.5 ± 4.1 ng/mL, *p* = 0.00001).

The follow-up analysis revealed a significant decrease in Gal-3 concentration in patients with atheroslerotic lesions in the carotid arteries (14.7 ± 7.2 ng/mL vs. 9.9 ± 2.1 ng/mL, *p* = 0.02). This relationship was not observed in the group of patients without carotid atherosclerosis.

### 3.6. Gal-3 Concentration and Occurrence of Adverse Cardiovascular Events during Follow-up Period

Subjects rehospitalized during the observation (due to both cardiac and non-cardiac disorders) had significantly higher concentration of Gal-3 at baseline in an acute phase of MI vs. those who were not hospitalized during follow up (13.7 ± 7.3 ng/mL vs. 8.5 ± 4.5 ng/mL, *p* = 0.002; 15.9 ± 8.1 ng/mL vs. 10.9 ± 4.9 ng/mL, *p* = 0.02, respectively). During the follow up, Gal-3 concentration did not change in patients with a subsequent MI. Patients with no coronary events during observation had a statistically significant decrease in Gal-3 concentration measured on the follow-up visit (baseline: 12.4 ± 7.9 ng/mL vs. follow up: 8.8 ± 2.5 ng/mL, *p* = 0.0002). Moreover, a significant decrease in Gal-3 concentration on the follow-up visit vs. baseline assessment was observed in patients who did not require cardiovascular intervention during follow up (re-PCI: 12.3 ± 7.1 ng/mL vs. 8.6 ± 2.4 ng/mL, *p* = 0.0005; coronary artery bypass grafting (CABG): 12.4 ± 7.5 ng/mL vs. 8.7 ± 2.5 ng/mL, *p* = 0.0001). Patients who had a stroke during the follow up did not show a significant decrease in Gal-3 concentration, whereas patients without a history of stroke revealed a statistically significant reduction in Gal-3 concentration on the follow-up visit (12.2 ± 6.7 ng/mL vs. 8.7 ± 2.4 ng/mL, *p* = 0.0002).

**Figure 1:**
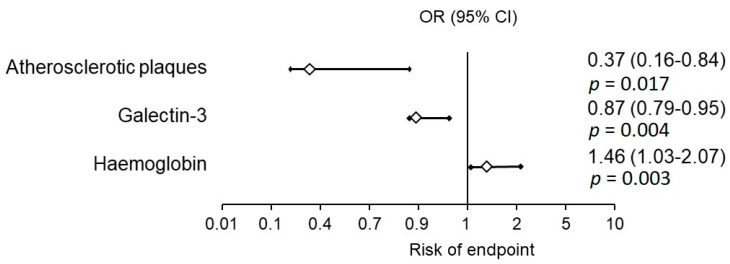
Odds ratio (OR) of the risk of the composite endpoint occurrence. CI - confidence interval.

On the basis of the receiver operating curve (ROC), the Gal-3 level ≥9.2 ng/mL has been assigned as a cut-off value with a high specificity (91%) but low sensitivity (50%) (*p* = 0.0005, area under the curve (AUC) = 0.684, 95% confidence interval (CI) = 0.581–0.788) for the occurrence of adverse cardiovascular events in MI patients during the follow up (see Figure 1).

**Figure 2:**
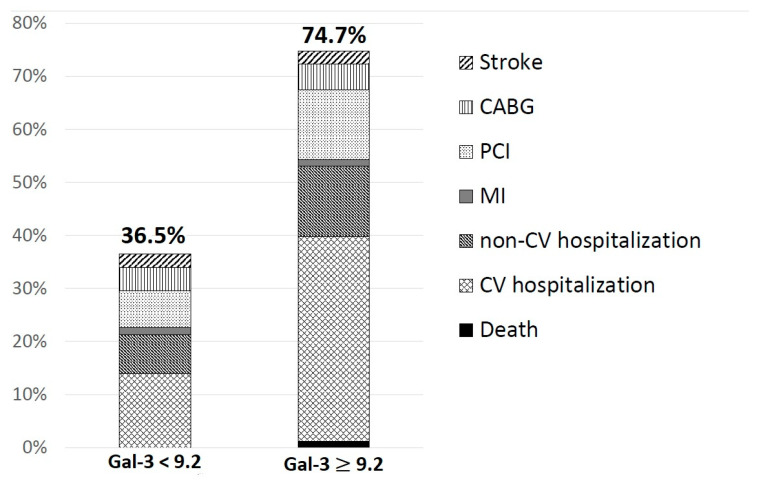
The frequency of adverse events due to galectin-3 concentration. CABG—coronary artery bypass grafting, CV—cardiovascular, Gal-3—galectin-3, MI—myocardial infarction, PCI—percutaneous coronary interventions.

In the group of patients with no endpoints: subsequent MI, re-PCI, CABG or stroke, a statistically significant decrease of Gal-3 concentration during observation was shown (Table 3). The univariate analysis, with parameters affecting frequency of the composite endpoint occurrence, included the presence of an atheromatous plaque in carotid arteries (*p* = 0.017, odds ratio (OR) = 0.37, 95% CI 0.16–0.84), Gal-3 concentration (*p* = 0.004, OR = 0.87, 95% CI 0.79–0.95) and haemoglobin concentration (*p* = 0.03, OR = 1.46, 95% CI 1.03–2.07). In the multivariate analysis, only Gal-3 concentration at discharge >9.2 ng/mL increased nine times the risk of the composite endpoint occurrence (*p* = 0.0005, OR = 9.47, 95% CI 2.60–34.45) (Figure 2). The increase of Gal-3 concentration of 1 ng/mL caused an increase of the composite endpoint occurrence by 14% (*p* = 0.004, OR = 1.145, 95% CI 1.04–1.26).

### 3.7. Carotid Ultrasonography Parameters

#### 3.7.1. At Baseline

The occurrence of atherosclerotic plaques was found only in the study group and not observed in the group of healthy volunteers (Table 4). The presence of atheromatous plaques in CCA was observed in 12 patients (pts.) (10.9%), while in CB it was observed in 87 pts. (79.1%).

#### 3.7.2. On the Follow-Up Visit

The presence of atheromatous plaques was observed in CCA in 10 pts. (9.1%), in CB in 69 pts. (62.7%); plaques in CCA, *p* = 0.5; in CB, *p* = 0.003, respectively (Wilcoxon signed-rank test).

## 4. Discussion

Gal-3 is a prognostic biomarker that has been studied in heart failure. The role of Gal-3 in the process of atherosclerosis is not fully recognized. There are limited data on this biomarker in the ACS population. In addition, some authors suggest that Gal-3 does not appear to have a promising role for assessing the severity of CAD [13]. Our present research revealed that in the study group of MI patients, the Gal-3 concentration was significantly higher than in the group of patients without symptomatic CAD. Tsai et al. [11] also demonstrates that the circulating level of Gal-3 was significantly higher in STEMI patients than in healthy control subjects. However, the role of the elevated Gal-3 level in an acute phase of MI remains debatable. Experimental studies suggest that this molecule actively contributes to the reparative processes in the infarcted area during the first hours of ischemia, which is essential for the maintenance of ventricular function after MI. On the other hand, in the later phase, Gal-3 might support the transition from acute to chronic inflammation and trigger cardiac fibrosis, leading to adverse ventricular remodeling and further heart failure development [17]. Our observation revealed that concentrations of Gal-3 correlated significantly with the presence of atheromatous plaques in carotid arteries, which were consistent with the results obtained by other researchers [18]. It should be noted that for the first time we have observed a reduction of Gal-3 concentrations in patients with atherosclerotic lesions in carotid arteries within the post-MI follow up. It was associated with a significant regression of atheromatous plaques diameter in CB observed in ultrasonography on the follow-up visit as compared to the baseline examination. We presume that this phenomenon results from the statins therapy implemented for all patients after a myocardial infarction. However, further research is needed to support this hypothesis. Kadoglou et al. proved that long-term statin treatment may induce an increase of an intraplaque Gal-3 concentration mediating plaque stabilization [19]. Gal-3 levels were higher in patients with a large artery atherosclerotic stroke than in controls [20]. A similar correlation was observed in our research. In addition, other researchers confirmed that higher levels of serum Gal-3 were independently associated with an increased risk of death or a major disability after a stroke onset, suggesting that Gal-3 may be of prognostic value in poor outcomes of ischemic stroke [21]. However, according to other researchers, an increased Gal-3 was associated with the primary outcome, stroke recurrence and vascular events within one year after stroke only in patients with hyperglycemia, suggesting that Gal-3 may be an important prognostic factor for ischemic stroke patients with hyperglycemia [22]. Moreover, it should be highlighted that among the classic cardiovascular risk factors, only hyperlipidemia affected the Gal-3 concentration both at baseline (significantly higher values of Gal-3 in an acute phase of MI) and in follow-up observation. The concentration of Gal-3 in patients with hyperlipidemia decreased significantly during the observation period. Because almost 90% of patients took a statin during follow up, it can be presumed that this therapy had an effect on lowering Gal-3 levels during follow up in this patient subgroup. Some authors observed a lower concentration of Gal-3 in atherosclerotic plaques in mice treated with atorvastatin, suggesting that intraplaque Gal-3 expression reflects the degree of plaque inflammation [23]. Other researchers showed small dense low density lipoprotein cholesterol to be an independent determinant of Gal-3 concentration, but it was observed in patients with chronic kidney disease [24]. However, we observed that pharmacotherapy results in a decrease of Gal-3 concentration only in the group of patients with single-vessel coronary artery disease, while there were no beneficial effects of such treatment in patients with more advanced CAD. Other researchers have shown that diabetes also affects high concentrations of Gal-3 [25], which we did not confirm in our studies. It is clinically relevant that in the group of patients after MI, who had a Gal-3 concentration decrease during follow up, there were no adverse cardiovascular events (another MI, re-PCI, CABG or stroke) observed. According to the authors’ knowledge, this is the first analysis of such data in the literature. Only Di Tano et al. showed that in anterior STEMI patients, early post-angioplasty Gal-3 concentration may be useful for risk stratification. However, it was only a few months-long observation; in over 90% of patients with Gal-3 concentration below 16.8 ng/mL, there were no adverse cardiovascular events observed [26]. Tan et al. observed that individuals in the highest quartile of serum Gal-3 (>9.73 ng/mL) had significantly elevated risk for cardiovascular endpoints and all-cause mortality; however, it only concerned patients with type 2 diabetes [27]. Research by other authors concentrated only on the hospitalization period and revealed that the increase of Gal-3 concentration in serum was associated with the risk of in-hospital major cardiovascular events in patients with acute ST-elevation MI [28]. On the contrary, in the group of patients with stable coronary artery disease, the Gal-3 did not independently predict recurrent cardiovascular events after an adjustment for multiple risk factors [29]. Concerning a value of Gal-3 of 9.2 ng/mL, similar cut-off was also achieved in different diseases (level ≥10.3 ng/mL as an independent prognostic marker in pancreatobiliary cancer [30], 10.53 ng/mL in patients with atrial fibrillation [31] or a cut-off value of Gal-3 ≥3.17 ng/mL to predict breast cancer [32]).

## 5. Conclusions

The significant decrease of Gal-3 concentration was observed in the group of patients after AMI, who had no endpoints such as subsequent MI, re-PCI, CABG or stroke during long-term observation. The decrease of Gal-3 concentration in follow up correlated with a significant regression of the atherosclerotic plaques in carotid arteries observed in ultrasonography. The multivariate analysis revealed that only Gal-3 concentration higher than 9.2 ng/mL at discharge was associated with the risk of composite endpoint occurrence during long-term follow up.

## Figures and Tables

**Table 1:** Patient characteristics at the beginning of the study.

		STEMI Group (*n* = 66)	NSTEMI Group (*n* = 44)	Control Group (*n* = 100)	*p*-Value Control Group vs. MI Group	*p*-Value STEMI vs. NSTEMI Group
Age (y)		62.4 ± 9.7	64.2 ± 10.1	61.5 ± 7.9	NS	NS
Sex	women	6 p. (9.1%)	16 p. (36.4%)	34 p. (33.7%)	NS	*p* < 0.005
	men	60 p. (90.9%)	28 p. (63.6%)	67 p. (66.3%)	NS	*p* < 0.005
BMI (kg/m^2^)		27.1 ± 2.4	27.5 ± 3.5	26.9 ± 3.4	NS	NS
Smoking (*n*)		40 p. (60.6%)	26 p. (59.1%)	28 p. (28%)	*p* < 0.001	NS
Hypertension (*n*)		44 p. (66.7%)	27 p. (61.4%)	0 p.	-	NS
Diabetes t.2 (*n*)		12 p. (18.2%)	5 p. (11.4%)	0 p.	-	NS
Hyperlipidemia (*n*)		25 p. (37.9%)	19 p. (43.2%)	0 p.	-	NS
Systolic BP (mmHg)		144.1 ± 24.0	146.2 ± 25.0	132.8 ± 19.0	*p* < 0.05	NS
Diastolic BP (mmHg)		88.6 ± 15.6	88.8 ± 13.6	83.0 ± 8.0	NS	NS
Total cholesterol (mmol/L)		4.96 ± 1.0	4.91 ± 0.95	4.40 ± 0.9	*p* < 0.05	NS
LDL cholesterol (mmol/L)		3.19 ± 0.95	3.11 ± 0.89	2.74 ± 0.8	*p* < 0.05	NS
HDL cholesterol (mmol/L)		1.15 ± 0.3	1.2 ± 0.24	1.6 ± 0.35	*p* < 0.05	NS
TG (mmol/L)		1.59 ± 0.95	1.53 ± 0.87	1.42 ± 0.54	NS	NS
Glucose (mmol/L)		6.35 ± 1.4	5.8 ± 1.3	5.65 ± 1.1	*p* < 0.05	*p* < 0.05
Creatinine (µmol/L)		91.05 ± 28.0	79.6 ± 19.5	89.2 ± 14.8	NS	*p* < 0.05
GFR (mL/min)		82.7 ± 25.6	87.1 ± 23.7	109.5 ± 30.0	*p* < 0.01	NS
Haemoglobin (mmol/L)		8.7 ± 0.8	8.5 ± 0.7	8.9 ± 1.0	NS	NS
EF (%)		45.9 ± 9.5	49.7 ± 10.1	55.0 ± 12.5	*p* < 0.01	NS (*p* = 0.05)
1-vessel disease (*n*)		25 p. (37.9%)	15 p. (34.1%)	-	-	NS
2-vessel disease (*n*)		15 p. (22.7%)	7 p. (15.9%)	-	-	NS
3-vessel disease (*n*)		25 p. (37.9%)	17 p. (38.6%)	-	-	NS

BMI—body mass index, BP—blood pressure, EF—ejection fraction, GFR—glomerular filtration rate, HDL—high density lipoprotein, LDL—low density lipoprotein, MI—myocardial infarction, NS—not statistically significant, NSTEMI—non-ST-elevation myocardial infarction, p.—patients, STEMI—ST-elevation myocardial infarction, TG—triglycerides.

**Table 2:** Clinical characteristics of the study group during follow up.

Clinical Characteristics of the Study Group	STEMI Patients (*n* = 66)	NSTEMI Patients (*n* = 44)	*p*-Value
CCS class	0.5 ± 0.8	0.6 ± 0.9	*p* = 0.5
NYHA class	1.5 ± 0.95	1.6 ± 1.0	*p* = 0.3
EF (%)	45.6 ± 9.9	49.5 ± 9.8	*p* = 0.04
Rehospitalization from cardiac causes	41 p. (60.3%)	27 p. (61.4%)	*p* = 0.9
Rehospitalization from non-cardiac causes	17 p. (25.0%)	12 p. (27.3%)	*p* = 0.8
Subsequent MI	0 p. (0%)	4 p. (9.1%)	*p* = 0.005
Re-PCI	18 p. (26.5%)	10 p. (22.7%)	*p* = 0.6
CABG	10 p. (14.7%)	5 p. (11.4%)	*p* = 0.6
Stroke	4 p. (5.9%)	5 p. (11.4%)	*p* = 0.3
Peripheral artery disease	10 p. (14.7%)	4 p. (9.1%)	*p* = 0.4
Carotid artery disease	2 p. (2.9%)	2 p. (4.5%)	*p* = 0.6
Smoking	23 p. (33.8%)	8 p. (18.2%)	*p* = 0.04
Treatment			
Aspirin	58 p. (85.3%)	38 p. (86.4%)	*p* = 0.9
Clopidogrel	8 p. (17.7%)	8 p. (18.2%)	*p* = 0.3
ACEIs	47 p. (69.1%)	23 p. (52.3%)	*p* = 0.04
ARBs	8 p. (11.8%)	6 p. (13.6%)	*p* = 0.8
Beta blockers	63 p. (92.6%)	39 p. (88.6%)	*p* = 0.5
MRAs	17 p. (25.0%)	9 p. (20.5%)	*p* = 0.6
Statins	62 p. (91.2%)	38 p. (86.4%)	*p* = 0.4
Diuretics	24 p. (35.3%)	19 p. (43.2%)	*p* = 0.4
Oral antidiabetic	15 p. (22.1%)	8 p. (18.2%)	*p* = 0.6
Insulin	6 p. (8.8%)	4 p. (9.1%)	*p* = 0.9
Oral anticoagulant	6 p. (8.8%)	3 p. (6.8%)	*p* = 0.5

ACEIs—angiotensin-converting enzyme inhibitors, ARBs—angiotensin II receptor antagonists, CABG—coronary artery bypass grafting, CCS—Canadian Cardiovascular Society, EF—ejection fraction, MI—myocardial infarction, MRAs—mineralocorticoid receptor antagonists, NSTEMI—non-ST-elevation myocardial infarction, NYHA—New York Heart Association, p.—patients, PCI—percutaneous coronary interventions, STEMI—ST-elevation myocardial infarction.

**Table 3:** Galectin-3 concentration at baseline and on the follow-up visit in the subgroup of patients without endpoints during observation.

No Endpoints during Follow Up	Gal-3 Concentration (ng/mL) at Baseline	Gal-3 Concentration (ng/mL) on the Follow-Up Visit	*p*-Value
Subsequent MI	10.8 ± 6.1	8.8 ± 2.4	*p* = 0.03
Re-PCI	10.6 ± 5.8	8.6 ± 2.4	*p* = 0.04
CABG	11.1 ± 7.5	8.7 ± 2.5	*p* = 0.016
Stroke	10.7 ± 5.9	8.7 ± 2.3	*p* = 0.03

CABG—coronary artery bypass grafting, Gal-3—galectin-3, MI—myocardial infarction, PCI—percutaneous coronary interventions.

**Table 4:** Carotid ultrasonography parameters in the study group (myocardial infarction patients) and in the control group (healthy volunteers).

	MI Group at Baseline (*n* = 110)	MI Group on the Follow-Up Visit	*p*-Value	Control Group (*n* = 100)	*p*-Value (MI Patients vs. Control)
Plaque occurrence in CCA	12 p. (10.9%)	10 p. (9.1%)	NS	0	*p* = 0.000001
Plaque occurrence in CB	87 p. (79.1%)	69 p. (62.7%)	*p* = 0.003	0	*p* = 0.000001

CB—carotid bulb, CCA—common carotid artery, MI—myocardial infarction, NS—not statistically significant, p.—patients.
